# Depression and Obstructive Sleep Apnea (OSA)

**DOI:** 10.1186/1744-859X-4-13

**Published:** 2005-06-27

**Authors:** Carmen M Schröder, Ruth O'Hara

**Affiliations:** 1 Department of Psychiatry and Behavioral Sciences, Stanford University School of Medicine, Stanford, CA 94305-5550, USA

**Keywords:** sleep apnea, OSA, sleep disordered breathing, mood, affective disorders

## Abstract

For over two decades clinical studies have been conducted which suggest the existence of a relationship between depression and Obstructive Sleep Apnea (OSA). Recently, Ohayon underscored the evidence for a link between these two disorders in the general population, showing that 800 out of 100,000 individuals had both, a breathing-related sleep disorder and a major depressive disorder, with up to 20% of the subjects presenting with one of these disorders also having the other. In some populations, depending on age, gender and other demographic and health characteristics, the prevalence of both disorders may be even higher: OSA may affect more than 50% of individuals over the age of 65, and significant depressive symptoms may be present in as many as 26% of a community-dwelling population of older adults.

In clinical practice, the presence of depressive symptomatology is often considered in patients with OSA, and may be accounted for and followed-up when considering treatment approaches and response to treatment. On the other hand, sleep problems and specifically OSA are rarely assessed on a regular basis in patients with a depressive disorder. However, OSA might not only be associated with a depressive syndrome, but its presence may also be responsible for failure to respond to appropriate pharmacological treatment. Furthermore, an undiagnosed OSA might be exacerbated by adjunct treatments to antidepressant medications, such as benzodiazepines.

Increased awareness of the relationship between depression and OSA might significantly improve diagnostic accuracy as well as treatment outcome for both disorders. In this review, we will summarize important findings in the current literature regarding the association between depression and OSA, and the possible mechanisms by which both disorders interact. Implications for clinical practice will be discussed.

## Depression in OSA

### Definition and prevalence of OSA

OSA is by far the most common form of sleep disordered breathing and is defined by frequent episodes of obstructed breathing during sleep. Specifically, it is characterized by sleep-related decreases (hypopneas) or pauses (apneas) in respiration. An obstructive apnea is defined as at least 10 seconds interruption of oronasal airflow, corresponding to a complete obstruction of the upper airways, despite continuous chest and abdominal movements, and associated with a decrease in oxygen saturation and/or arousals from sleep. An obstructive hypopnea is defined as at least 10 seconds of partial obstruction of the upper airways, resulting in an at least 50% decrease in oronasal airflow.

Clinically OSA is suspected when a patient presents with both snoring and excessive daytime sleepiness (EDS) [1,2]. The diagnosis of OSA is confirmed when a polysomnography recording determines an Apnea-Hypopnea-Index (AHI) of > 5 per hour of sleep [3]. Even if cutoff points have never been clearly defined, an AHI of less than 5 is generally considered being normal, 5–15 mild, 15–30 moderate and over 30 severe OSA.

The prevalence of OSA is higher in men than in women. OSA is found in all age groups but its prevalence increases with age. In children, the prevalence of OSA is less well defined and has been estimated to be 2–8% [4]. In subjects between the ages of 30 to 65 years, 24% of men and 9% of women had OSA [5]. Among subjects over 55 years of age, 30–60% fulfil the criterion of an AHI > 5 [6-8]. In a population of community-dwelling older adults, 70% of men and 56% of women between the ages of 65 to 99 years have evidence of OSA with a criterion of AHI > 10 [9].

The abnormal respiratory events which are the hallmark of OSA are generally accompanied by heart rate variability and arousals from sleep, with frequent arousals being the most important factor resulting in EDS. With regards to sleep architecture, we find a significant increase in light sleep stage (mainly stage 1) at the expense of deep slow wave sleep (stages 3 and 4) and REM sleep. Slow wave sleep is sometimes even completely abolished. However clinically, patients are often not aware of this repetitive sleep interruption (with sometimes hundreds of arousals during one night), but simply do not feel restored in the morning. Other nocturnal symptoms can include restlessness, nocturia, excessive salivation and sweating, gastroesophageal reflux, as well as headache and dry mouth or throat in the morning on awakening.

The extent to which daytime functioning is affected generally depends on the severity of OSA. Symptoms other than EDS which greatly impact daytime functioning are neuropsychological symptoms such as irritability, difficulty concentrating, cognitive impairment, depressive symptoms, and other psychological disturbances. Thus, OSA can easily mimic symptoms of a major depressive episode.

### Correlation studies of OSA and depression

Among the first studies investigating the relation between OSA and depression, Guilleminault et al. [10] reported that 24% of 25 male patients with OSA had previously seen a psychiatrist for anxiety or depression, and Reynolds et al. [11] showed that around 40% of 25 male OSA patients met the research diagnostic criteria for an affective disorder, with a higher risk of depression in those patients who were sleepier during the day. Similarly, Millmann et al. observed that 45% of his 55 OSA patients had depressive symptoms on the Zung Self-Rating Depression Scale, with the group scoring higher for depression also having a significantly higher AHI [12]. Whereas only 26% of OSA patients described themselves as currently depressed, 58% fulfilled DSM-III criteria for major depression of four or more depressive symptoms [13]. Others observed increased depression scores on the Minnesota Multiphasic Personality Inventory (MMPI) in patients with OSA [14,15]. Indeed, Ramos Platon et al. found elevations in several MMPI scales in 23 OSA patients (moderate to high severity) compared to 17 controls [16]. Aikens et al. [17] showed that 32% of their OSA patients had elevated depression scores on the MMPI and in the same series of studies, there were twice as many OSA patients with elevated depression scores than age and sex matched primary snorers [18]. However, the percentage of depressive symptoms was not significantly different when compared to patients with other primary sleep disorders, such as periodic limb movements during sleep (PLMS) [19]. Most recently, in an epidemiological study of 18,980 subjects representative of the general population in their respective countries (UK, Germany, Italy, Portugal, and Spain) and assessed by cross-sectional telephone survey, Ohayon determined that 17.6% of subjects with a DSM-IV breathing-related sleep disorder diagnosis also presented with a major depressive disorder diagnosis, and vice versa [20]. This correlation persisted after controlling for obesity and hypertension.

In contrast to the numerous studies observing a positive correlation between OSA and depression, some investigations found no association between both disorders. In a 5-year longitudinal study, Phillips et al. did not find any significant depressive symptoms in elderly patients with a relatively mild OSA (AHI>5/h), when compared to a control group without OSA (AHI<5/h) [21]. However, there are multiple limitations to this study, besides a relatively small sample size for group comparisons and a non-representative study population. OSA was only assessed at baseline, but not repeated at the five-year follow-up, i.e. neuropsychological data were compared between two groups based on OSA status five years earlier. Second, OSA severity was mild even in the OSA group. Third, the groups differed significantly by age, with the OSA group being older than the control group. Finally, the attrition rate over the five years was very high with only 42 out of the initial 95 subjects completing the follow-up assessment. In another large-scale study, Pillar and Lavie did not observe any association between respiratory disturbances and Symptom Check List 90 in 2,271 predominantly male patients assessed for OSA [22]. However, the SCL-90 questionnaire was developed as a screening tool for psychiatric patients, and not for a normal study population. Therefore, it might be a less sensitive tool with regards to milder forms of mood disturbances than other scales. Interestingly, Pillar and Lavie observed that among the minority of women in this study, those with severe OSA had higher depression scores than those with mild OSA. Bardwell found that other factors such as age, body mass index (BMI) and hypertension accounted for the correlation between sleep parameters and total mood disturbances in 72 OSA patients when compared to 40 controls [23]. However, the chosen cutoff point to distinguish between OSA and the control group in this study was relatively high (AHI of 15/h), thus subjects with a mild OSA were probably included in the control group.

In sum, the majority of studies to date report an association between depression and OSA, but methodological considerations render the comparison between investigations difficult. Some of the mixed findings among studies can be explained by differences in sample size, study population, gender distribution, age and AHI cut-off in relation to age, as well as variability in terms of the questionnaires and scales used to assess depressive symptomatology. Given the heterogeneity of these data and considering the numerous confounding factors, future longitudinal studies of patient populations are required to better understand the relation between both disorders.

### Treatment Studies for OSA: reversibility of depressive symptoms?

The gold standard treatment for moderate to severe cases of OSAS is continuous or bilevel positive airway pressure (CPAP/BiPAP) which mechanically maintains the upper airways space open during sleep via the administration of ambient air with a certain pressure. The minimum necessary pressure level has to be titrated individually for each patient [24]. Other treatments, especially for mild cases of OSA, include weight loss, dental devices (which advance the tongue or mandible to increase posterior airway space) or upper airway surgery (e.g. combined tonsillectomy/ adenoidectomy, nasal reconstruction, and uvulopalatopharyngoplasty). Different upper airway surgical procedures can be used for particular cases with craniofacial abnormalities [25].

Overall, CPAP treatment studies for OSA and its effect on depressive symptoms have yielded controversial findings. Derderian et al. [26] compared results on the Profile of Moods Questionnaire before and after 2 months of CPAP treatment in an OSA group (n = 7) and showed a significant drop in Total Mood Disturbance. This improvement was correlated with an increase in slow-wave sleep. Those patients in the study of Millmann et al. who received CPAP displayed a significant decrease in their Zung Depression Scale scores [12]. Similarly, Engleman et al. reported an improvement in a comprehensive battery of mood and cognitive assessment scales after 4 weeks of CPAP treatment in 32 patients with moderate OSA [27] as well as in 16 patients with a mild OSA [28]. Means et al. [29] showed an improvement on Beck Depression Inventory (BDI) depression scores after 3 months of treatment in 39 OSA patients, and Sanchez et al. [30] confirmed lower BDI scores after 1 and 3 months of CPAP therapy in 51 OSA patients. Ramos Platon et al. [16] underscored the progressive improvement in depression scores on the MMPI scale over the first year of treatment. A systematic review on the influence of CPAP on neurobehavioral performance of patients with OSA also supported the clinical perspective that typically depressive symptoms remit together with EDS under CPAP therapy [31].

Among the negative studies on CPAP therapy and its effect on depression, Borak et al. [32] did not observe any improvement in emotional status after 3 and 12 months of CPAP therapy in 20 patients with severe OSA, similar to Munoz et al. [33] who also did not show improvement of BDI scores in 80 subjects with severe OSA after 12 months of CPAP. Using subtherapeutic CPAP as the placebo control, Yu et al. [34] and Henke et al. [35] found no difference in improvement on depression scores between the treatment and the control group, over a short treatment duration (1–3 weeks). However, whereas Borak, Munoz and Henke do not find any effect of CPAP therapy on mood, Yu observed a positive effect on mood of both CPAP therapy and the subtherapeutic CPAP control group.

Intriguingly, there are no systematic differences with regards to the sample size, the initial severity of OSA or the duration of CPAP therapy which might explain the differences between studies observing an improvement after CPAP therapy and those who did not. Several issues have to be considered: First, it is difficult to design a good control ("placebo") condition for CPAP treatment. "Sham-CPAP" which uses insufficient positive airway pressure as a placebo condition (1 – 2 cm H_2_0), is now used more frequently. Two of the negative studies employed this method for their control group, which raises the possibility that the previously observed positive effects of CPAP on mood may have been a placebo effect. Second, compliance to CPAP treatment is problematic, because patients have to wear a nasal or even an oranasal device during the entire night. The compliance may even be particularly decreased in depressed patients. Indeed, Edinger et al. [36] reported a positive correlation between lower depression scores on the MMPI prior to treatment and CPAP compliance at 6 months of treatment in 28 patients. However, Lewis et al. [37] did not find any association between baseline depression scores and subsequent CPAP use for the first month of treatment. The most important factor to explain the differences among these studies may be the variability in the severity of initial depressive symptoms. Whereas the severity of OSA itself does not seem to have a differential impact on mood improvement after CPAP therapy, the severity of depressive symptoms associated with OSA may impact response to CPAP treatment. As Millmann indicates, OSA patients with more severe mood symptoms responded better to CPAP treatment, whereas patients with less severe or no mood symptoms actually had less benefit from CPAP therapy [12]. However, all negative treatment studies either excluded subjects suffering from a major depressive disorder, or their depression scores were even at baseline in a normal range (baseline values: mean BDI of 7.5 in [32], mean depression score on POMS scale of 12.5 in [34], mean BDI of 8 in [33], and no information given on assessed GDS scores in [35]). Future studies should seek to include OSA patients with a broader range of depressive symptoms in treatment studies, to investigate whether CPAP might have a better effect on mood in more depressed OSA patients.

## OSA in depression

Compared to the large number of studies investigating depressive symptomatology in OSA patients, far fewer studies have focused on the screening for OSA in a primarily depressed study population. In one of the few investigations of the prevalence of OSA in a depressed cohort, Reynolds et al. found, in a small sample of 17 older patients with major depression, that 17.6% also had an OSA syndrome, compared to 4.3% of 23 healthy elderly controls [38]. This suggests that OSA might be an important confounding factor for studies on mood disorders in general, as its presence is not routinely determined in either research studies examining mood or clinical settings. However, many more studies are required to assess the prevalence of OSA in primarily depressed patients, particularly as it can be suspected from existing studies that OSA is greatly underdiagnosed in this patient population.

Clinically, this is of particular concern, as sedative antidepressants and adjunct treatments for depression may actually exacerbate OSA. Notably hypnotics prescribed to treat depression-related insomnia might further decrease the muscle tone in the already functionally impaired upper airway dilatator muscles, blunt the arousal response to hypoxia and hypercapnia as well as increase the arousal threshold for the apneic event, therefore increasing the number and duration of apneas [39,40]. These effects might differ depending on the patient population and the severity of OSA. Older depressive subjects are of primary concern: both, frequency of OSA and depressive symptoms increase with age, as do prescription and consumption of sedative psychotropic medication. Pharmacologic treatment of depression and depression-related insomnia in this age group should therefore routinely consider the potential presence of a concomitant OSA.

Finally, as Baran and Richert point out, the diagnosis of a mood disorder in the presence of OSA has its very own challenges [41]. Considering the DSM-IV definitions [42], it could either be viewed as a mood disorder due to a general medical condition, or classified as an adjustment disorder with depressed mood, due in particular to EDS and its debilitating consequences on the patients' daytime functioning. The identification of pathophysiological features that allow distinction between OSA and depression might assist with such diagnostic issues.

## Sleep architecture in depression and OSA

Both depression and OSA have been well characterized with regards to their sleep architecture. Typically, for major depression, polysomnography (PSG) findings confirm the patients' complaints of insomnia, notably difficulties falling asleep (PSG: increase in sleep latency), frequent awakenings during the night and early morning awakenings (PSG: idem) as well as non-refreshing sleep (PSG: decrease in slow wave sleep). PSG furthermore reveals a shortened REM latency, i.e. the first episode of REM sleep appears earlier than usual, with an increase in total percentage of REM sleep during the night, as well as in its eye movement density (referred to as REM sleep disinhibition) [43]. On the other hand, the sleep of patients with OSA is fragmented, and contains a lot of transitional sleep stages (stage 1) at the expense of REM sleep and particularly of slow wave sleep (stages 3 and 4) [44,45]. At least two studies have investigated sleep architecture at the interplay of OSA and depression or depressive symptoms. Reynolds et al. stated that, in contrast to the sleep EEG of depressed patients which characteristically shows a shorter latency of REM sleep, sleep apnea patients with depression displayed an increase in REM latency [11]. Bardwell et al. compared a group of 106 patients with and without OSA with regards to their sleep architecture. Depressed patients who also had OSA displayed a decrease in sleep latency when compared to the depressed group without OSA; and OSA subjects with depressive symptoms had a higher percentage of REM sleep than OSA subjects without depression [46]. Rather than distinguishing a primary depressive illness from an organic affective syndrome related to OSA [11], however, the aforementioned polysomnographic results underscore how both disorders interplay, thus confounding EEG findings characteristic for each disorder.

## Possible mechanisms underlying the association between depression and OSA

### Sleep fragmentation and hypoxemia

The two main factors suspected to be responsible for depressive symptoms in OSA are sleep fragmentation and oxygen desaturation during sleep. Sleep fragmentation is a direct consequence of the recurrent microarousals associated with the apneas and hypopneas, and the nocturnal hypoxemia is due to the intermittent drops in oxygen saturation caused by the respiratory events [47]. Sleep fragmentation is the primary cause of EDS in OSA patients, and is suggested to result in the depressive symptomatology in OSA. This last perspective gains support from the finding that EDS as measured by the Epworth Sleepiness Scale (ESS) and the Maintenance of Wakefulness Test (MWT) was found to be correlated with higher depression scores on the Hospital Depression Scale (HAD-D) in 44 patients with OSA [48]. Furthermore, a Canadian study on 30 OSA patients showed a significant correlation between the severity of psychological symptoms on SCL-90 and less total sleep time, as well as percentage of wake time after sleep onset and ESS scores [49]. With respect to hypoxemia, Engleman et al. noted in a recent review that the effect size of cognitive impairment in OSA correlated highly with severity of hypoxic events, ranging from .3 standard deviations for milder levels of AHI to 2–3 standard deviations for higher levels of AHI [50]. Recently, preliminary imaging data suggests that hypoxemia related to OSA might also play a role in impacting mood. Cerebral metabolic impairment resulting from recurrent nocturnal hypoxemia in OSA have had previously been observed in several imaging investigations on OSA [51-53]; independently, white matter hyperintensities (WMH) have been linked to depressive symptomatology in studies on affective disorders [54-58]. Aloia et al. reported in a small sample of older patients with OSA more subcortical WMH in the brain MRI of patients with a severe OSA as compared to those with minimal OSA, and a tendency for a positive correlation between these subcortical hyperintensities and depression scores on the Hamilton Depression Scale [59].

### Neurobiology of depression and upper airway control in OSA: the role of serotonin

The high comorbidity of OSA and depression also suggests that both disorders may share a common neurobiological risk factor. On the neurotransmitter level, the serotoninergic system has a central role as a neurobiological substrate underlying impairments in the regulations of mood, sleep-wakefulness cycle, and upper airway muscle tone control during sleep. Depression is associated with a functional decrease of serotoninergic neurotransmission, and is mostly responsible for the alterations in sleep as outlined above [60].

The physiopathology of OSA involves numerous factors, among whose the abnormal pharyngeal collapsibility during sleep is one of the most compelling. Serotonin delivery to upper airway dilatator motor neurons has been shown to be reduced in dependency of the vigilance state [61]. This leads to reductions in dilator muscle activity specifically during sleep, which may contribute to sleep apnea. However, whereas the role of serotonin in mood disorders has been largely documented, its involvement in the pathophysiology of sleep apnea remains to be clarified. Interestingly, molecules increasing 5-HT neurotransmission such as the Serotonin reuptake inhibitors (SSRI) are widely prescribed antidepressant molecules that are suggested to similarly improve the apnea hypopnea index in OSA. Serotoninergic drugs such as fluoxetine, protryptiline and paroxetine have already been tested for OSA, with limited success and numerous adverse effects [61]. Several 5-HT receptor ligands and bi-functional molecules are under development, which may in the future be able to target both, the depressive syndrome and OSA.

### Shared risk factors

OSA and depression share common risk factors, which may partly explain their high comorbidity in the general population. Very frequently in studies of the impact of OSA on cognitive and psychological functioning, a conglomerate of disorders is shown to contribute to the overall neuropsychological outcome. Therefore, the presence of a polypathology often associated with OSA, such as obesity, cardiovascular disease, hypertension and diabetes, should increase the suspicion of an underlying or coexisting OSA in a depressed patient.

Both, depression and OSA, have independently been shown to be associated with metabolic syndrome, and also with the development of cardiovascular disease [62,63]. The association between depression and metabolic syndrome has been suggested to be reciprocal [64], and a priori not attributable to genetic factors as twin studies revealed [65]. In particular, insulin resistance (IR) has been suggested to contribute to the pathophysiology of depressive disorder and has been proposed to subserve the association between depression and cardiovascular disease [66]. Similarly, OSA has been observed to be independently associated with the cardiovascular risk factors comprising metabolic syndrome [67], in particular IR [68]. The magnitude of this association has even led researchers to suggest that metabolic syndrome should encompass OSA [69].

Although OSA and depression share these common risk factors, there are currently no studies available which have investigated the issue of antecedent or consequence in the relationship between depression, OSA and metabolic syndrome, and if and how these three highly prevalent disorders may interact to exacerbate the risk for cardio – and cerebrovascular morbidity and mortality.

## Clinical application

As a consequence of the complex relationship between depression and OSA, the assessment of a patient's individual sleep history should be included in the standard psychiatric clinical interview, and specifically in the assessment of a depressive syndrome. A clinician should suspect OSA particularly in those depressed patients who present with its cardinal symptoms, namely, 1) loud snoring or intermittent pauses in respiration, as witnessed by a bed partner, associated with 2) excessive daytime sleepiness (EDS). Given that patients often deny the latter, standardized questionnaires such as the Epworth Sleepiness Scale (ESS) [70] or the Functional Outcome Sleep Questionnaire (FOSQ) [71] are useful tools to assess EDS. The ESS asks the patients to rate their chances to fall asleep during periods of relaxation or inactivity (such as reading, watching television), but also in more active settings (driving a car, sitting and talking to someone). EDS is by far the most frequent daytime symptom of OSA, whereas nocturnal symptoms include restlessness, nocturia, excessive salivation and sweating, gastroesophageal reflux, as well as headache and dry mouth or throat in the morning on awakening. Furthermore, the clinical picture frequently includes obesity and hypertension, and, in those patients who are not obese, special facial abnormalities which narrow the upper airway, such as retrognathia or micrognathia.

However, it should be kept in mind that OSA may not be immediately apparent, but might present in an atypical fashion, with irritability, tiredness, disrupted sleep, difficulty concentrating, difficulties accomplishing tasks and generally decreased psychomotor performance [12]. Women are more likely to present with these symptoms [22,72,73], and have been suggested to be particularly underdiagnosed because of their atypical symptoms [74]. The importance of the sleep-wake complaints in a patient's depressive profile, and the onset of those complaints prior to the development of the depressive psychopathology should draw the clinician's attention to a potential underlying or coexisting OSA [75].

Third, particular attention should be paid to depressive patients who are resistant to treatment. In this case, OSA should be excluded as a major underlying contributing factor [76], as treatment of OSA could improve not only the compliance to pharmacological antidepressant treatment, but also the treatment response rate for depression [77]. Fourth, comorbid disorders of OSA may also catch the attention of the treating psychiatrist. In addition to the outlined association with the metabolic syndrome, Farney et al. observed that the likelihood of OSA increased significantly when either antihypertensive or antidepressant medications had been prescribed [78].

Depressed patients with a suspected OSA should be referred to a sleep disorders center for evaluation by nocturnal polysomnography, to confirm the diagnosis of OSA or the presence of other forms of sleep disordered breathing, such as the upper airway resistance syndrome [79]. This is of particular importance, as some of the adjunct treatments to the current pharmacological treatment of depression may actually exacerbate the condition.

If the diagnosis of OSA has been established in a depressed patient, and treatment has been initiated, close follow-up of the improvement of the depressive symptoms might give some indications as to the extent to which the presence of OSA may have contributed to the depressive symptomatology. However, as Baran and Richert point out [41], the aforementioned diagnostic challenge of a depressive syndrome in the presence of OSA currently remains unresolved.

On the other hand, systematic assessment of depressive symptoms with standardized clinical questionnaires in OSA patients is generally part of the evaluation process in all major sleep disorder centers. However, as these questionnaires have not been specifically designed to assess depression in OSA patients [80], they might be inappropriate to assess depression in this population, given that it is still unclear if OSA and depression display a true comorbidity or only share similar symptoms [41]. Typically, patients with severe depressive symptoms should be referred to a psychiatrist, particularly if such symptoms do not regress or if fatigue lingers after efficient treatment of OSA [81].

## Conclusion

Recent studies underscore the existence of a complex relationship between depression and OSA in terms of clinical presentation, underlying pathophysiology and treatment. It should incite the treating psychiatrist to be highly aware of a possibly underlying or coexisting OSA in depressed patients. Up to 20% of all patients presenting with a diagnosed depressive syndrome may also have OSA, and vice versa. This relationship might vary widely, depending on age, gender, AHI cut-off and general demographic and health characteristics of the population under investigation. Future clinical research in this area should specifically examine depressed patient populations, taking into account the different sub-type of mood disorders, and investigate a broader range of depressive symptomatology in OSA patients. Basic research should further investigate the causal relationship between depression and OSA, as well as the potential mechanisms by which both disorders may interact.

## Competing interests

The author(s) declare that they have no competing interests.

## Authors' contributions

CS and ROH both reviewed the existing literature and drafted the manuscript. Both authors approved the final manuscript.
